# Procainamide Inhibits DNA Methylation and Alleviates Multiple Organ Dysfunction in Rats with Endotoxic Shock

**DOI:** 10.1371/journal.pone.0163690

**Published:** 2016-09-23

**Authors:** Chih-Chin Shih, Mei-Hui Liao, Tsan-Seng Hsiao, Hiong-Ping Hii, Ching-Hui Shen, Shiu-Jen Chen, Shuk-Man Ka, Yung-Lung Chang, Chin-Chen Wu

**Affiliations:** 1 Graduate Institute of Medical Sciences, National Defense Medical Center, Taipei, R.O.C., Taiwan; 2 Department of Pharmacology, National Defense Medical Center, Taipei, R.O.C., Taiwan; 3 Department of Surgery, Chi Mei Medical Center, Tainan, R.O.C., Taiwan; 4 Department of Anesthesiology, Taichung Veterans General Hospital, Taichung, R.O.C., Taiwan; 5 School of Medicine, National Yang-Ming University, Taipei, R.O.C., Taiwan; 6 Department of Physiology, National Defense Medical Center, Taipei, R.O.C., Taiwan; 7 Department of Nursing, University of Kang-Ning, Taipei, R.O.C., Taiwan; 8 Department of Health Management for Elderly Society, University of Kang-Ning, Taipei, R.O.C., Taiwan; 9 Graduate Institute of Aerospace and Undersea Medicine, National Defense Medical Center, Taipei, R.O.C., Taiwan; 10 Department of Biochemistry, National Defense Medical Center, Taipei, R.O.C., Taiwan; Max Delbruck Centrum fur Molekulare Medizin Berlin Buch, GERMANY

## Abstract

Excessive inflammatory and oxidative stress lead to circulatory failure, multiple organ dysfunction, and high mortality in patients with sepsis. Microbial infection-induced DNA hypermethylation is associated with the augmentation of inflammation and oxidative stress. In our previous study, the antiarrhythmic drug procainamide inhibits the expression of DNA methyltransferase 1 (DNMT1) and diminishes IL-6 levels in rats with rhabdomyolysis. Thus, we further evaluated the effects of procainamide on the development of circulatory failure and multiple organ dysfunction in rats with endotoxic shock. Male Wistar rats were intravenously infused with saline or lipopolysaccharide (LPS) followed by procainamide administration. The changes of hemodynamics, blood glucose, biochemical variables, and plasma nitric oxide (NO) levels were analyzed during the experimental period. At the end of experiments, animal organs were also obtained for examining superoxide production, neutrophil infiltration, and DNA methylation status. Our results showed that LPS induced circulatory failure, multiple organ dysfunction, and high mortality rate in endotoxemic rats. Overt neutrophil infiltration and superoxide production, accompanied by the elevations of DNMT1 and 5-methylcytosine levels in the lung of endotoxemic rats were also observed. Treatment of endotoxemic animals with procainamide not only inhibited the increased levels of DNMT1 and 5-methylcytosine but also ameliorated neutrophil infiltration and superoxide production in the lung. In addition, the anti-inflammatory gene, IL27RA, was down-regulated in the LPS group and up-regulated in the LPS + Procainamide group. Procainamide also diminished IL27RA methylation in the lung of endotoxemic rat. Moreover, both DNMT inhibitors procainamide and hydralazine improved hypotension, hypoglycemia, and multiple organ dysfunction of LPS-treated rats. Thus, we suggest that the beneficial effects of procainamide could be attributed to the suppression of DNA methylation, neutrophil infiltration, superoxide production, and NO formation. It seems that this old drug may have new potential uses in infectious diseases, in particular, associated with endotoxemia.

## Introduction

Sepsis is a life-threatening disease triggered by the invasion of microbes and dysregulation of innate immune system [1]. Certain microbial toxins, such as lipopolysaccharide (LPS), are capable of engaging Toll-like receptors to activate the immune cells and other cell types. Excessive production of cytokines, reactive oxygen species (ROS), and nitric oxide (NO) causes systemic inflammatory response, redox imbalance, and arterial hypotension. These phenomena lead to progressive and irreversible multiple organ dysfunction and high mortality during sepsis [2]. However, so far inhibitors of these pathways are not able to fully recover patients or animals with sepsis. Thus, new therapeutic options for further improvement in outcome of sepsis are needed.

DNA methylation is an epigenetic mechanism that involves the conversion of cytosine in the CpG dinucleotide into methylcytosine for the regulation of gene expression [3, 4]. DNA methyltransferases (DNMTs) are responsible for establishing CpG methylation patterns by maintaining or de novo DNA methylation [5]. This epigenetic control in the host can be modified by many environmental factors such as exposure to pathogenic bacteria or viruses [6–8]. It has been demonstrated that DNMT activity, DNMT1 expression, and DNA methylation level are increased in uroepithelial cells after infection with uropathogenic Escherichia coli [9, 10]. In addition, recent studies reveal that LPS is a trigger for the elevation of DNA methylation to alter gene expression. Aberrant DNA methylation in rat lung tissues is observed in LPS-induced acute lung injury [11]. In addition, exposure to LPS significantly up-regulates the expression of (i) DNMT1 in macrophages and (ii) DNMT1, DNMT3A, DNMT3B, and methyl CpG binding protein in liver and spleen tissues [12–14]. Moreover, administration of LPS-treated macrophage cells with DNMT inhibitor 5-aza-2′-deoxycytidine (5-aza-dC) or DNMT1 RNAi significantly attenuates promoter hypermethylation of suppressor of cytokine signaling and diminishes the secretion of inflammatory cytokines [12]. These data suggest that pathogenic bacteria and its component are important mediators to interfere with DNA methylation.

DNMT inhibitors provide great chances for the development of efficient drugs to alleviate DNA hypermethylation in diseases. Nucleoside inhibitors of DNMTs, such as 5-aza-dC, have been widely used to reverse abnormal DNA methylation status [15–18]. However, nucleoside analogs could have myelotoxicity and incorporate into DNA that might lead to mutation of surviving cells [19–21]. Procainamide is one of the class 1A antiarrhythmic drugs used to treat a variety of atrial and ventricular dysrhythmias. Further investigation revealed that procainamide is also a non-nucleoside specific inhibitor of DNMT1, which can restore the expression of tumor suppressor genes silenced by DNA hypermethylation in cancer cells [22–25]. In addition, our previous study also showed that procainamide could improve the syndromes and complications of rhabdomyolysis through the inhibition of DNMT1 [26]. However, the therapeutic effects of procainamide on endotoxic shock have not been demonstrated. Therefore, we hypothesized that procainamide may be used as an effective demethylating drug against circulatory failure and multiple organ dysfunction that occur in endotoxic shock.

## Materials and Methods

### Ethics statement

Animal experiments were approved by the Institutional Animal Care and Use Committee of National Defense Medical Center (Taipei, R.O.C., Taiwan) (Permit Number: IACUC-12-131) and performed in adherence to the National Institutes of Health guidelines. Humane endpoints and euthanized animals prior to the end of the experiments were used in this study. We determined when the animals should be euthanized by the signs of weight loss, dyspnea, cyanosis, inappetence, extreme reluctance to stand, depression coupled with low body temperature, severe diarrhea, seizures, paralysis of one or more extremities. Overdose anesthetic (sodium pentobarbital) was the method of euthanasia in this study. The health of the rats were examined and monitored every 1 h, and there were no unexpected deaths in these cases. We used anesthesia (sodium pentobarbital) to reduce the distress and suffering of animals before any process that is potentially stressful or painful.

### Animals and experimental protocols

Male Wistar rats (10–12 weeks old, 260–300 g) were purchased from BioLASCO Taiwan Co (Taipei, R.O.C., Taiwan) and were guaranteed free of particular pathogens. Rats were anesthetized by intraperitoneal injection of sodium pentobarbital (50 mg/kg). Polyethylene catheters were placed in the right jugular vein for the administration of drugs and in the left carotid artery for the measurement of hemodynamics and blood sampling. After recovering to the normal condition overnight, the animals were randomly divided into four groups and treated with different agents intravenously (i.v.) as follows: (1) Control group was given vehicle (0.5 mL/kg of saline) at time 0; (2) Control + Procainamide group was given vehicle as in Control group and then given procainamide (50 mg/kg, infusion 30 min) at 1 h; (3) LPS group was given *Escherichia coli* lipopolysaccharide (LPS, 5 mg/kg) at time 0; (4) LPS + Procainamide group was given LPS (5 mg/kg) at time 0 and then given procainamide (50 mg/kg, infusion 30 min) at 1 h. The experiments were performed on pairs of rats and monitored for 6 h. In a separate experiment, effects of another DNMT inhibitor hydralazine [23, 25] on endotoxemic rats were also determined. Animals were randomly divided into four groups: (1) Control group was given vehicle at time 0; (2) Control + Hydralazine group was given vehicle as in Control group and then given hydralazine (0.3 mg/kg, infusion 15 min) at 0.5 h; (3) LPS group was given LPS at time 0; (4) LPS + Hydralazine group was given LPS at time 0 and then given hydralazine (0.3 mg/kg, infusion 15 min) at 0.5 h.

The arterial catheter was connected to a pressure transducer (P23ID, Statham, Oxnard, CA, USA) to measure mean arterial blood pressure (MAP) and heart rate (HR), which were recorded on a MacLab/4e poly-graph recorder (AD Instruments Pty Ltd., Castle Hill, Australia). After baseline hemodynamic parameters were monitored, animals were intravenously given norepinephrine (NE, 1 μg/kg) to examine the pressor responses. In order to normalize the baseline value of pressor responses to NE of all groups, the value of pressor responses to NE at time 0 was regarded as 100%. Blood samples were collected at baseline (i.e., time 0) and specified times (i.e., at 1, 2, 4, and 6 h) to examine the changes of blood glucose, hepatic function index (i.e., alanine aminotransferase [ALT]), renal function index (i.e., creatinine [CRE] and blood urea nitrogen [BUN]), cell injury index (i.e., lactate dehydrogenase [LDH]), and plasma nitric oxide (NO) concentration. Each volume of blood removed was immediately displaced by the injection of an equal volume of sterile saline. At 6 h after vehicle or LPS, the surviving rats were sacrificed by overdosed pentobarbital, and aortas, lungs, livers, and kidneys were immediately exercised to analyze superoxide production, histologic assessment, and the levels of DNMT1 and 5-methylcytosine. The survival rate was calculated during the experimental period.

### Measurement of blood glucose

Blood samples were drawn at baseline (i.e., time 0) and at 1, 2, 4, 6 h after vehicle or LPS. Ten microliters of whole blood was immediately used to analyze the glucose levels by a One Touch II blood glucose monitoring system (Lifescan, Milpitas, CA, USA).

### Quantification of organ function and injury

Arterial blood samples were collected and immediately centrifuged at 16,000 *g* for 2 min to obtain the plasma for measuring biochemical variables and NO levels. Sixty microliters of plasma was used to evaluate organ function and injury at baseline (i.e., time 0) and at 1, 2, 4, 6 h after vehicle or LPS. The following enzymes analyzed in the plasma were regarded as biochemical indicators of organ function and injury. For instance, liver dysfunction was assessed by measuring the increase in plasma levels of ALT. Renal dysfunction was assessed by the increases in plasma levels of CRE and BUN. In addition, the extent of organ injury was evaluated by the increase in plasma levels of LDH. All of the biochemical variables were analyzed by Fuji DRI-CHEM 3030 (Fuji Photo Film, Tokyo, Japan).

### Measurement of plasma NO concentration

Thirty microliters of plasma obtained at baseline (i.e., time 0) and at 1, 2, 4, 6 h after vehicle or LPS was de-proteinized by incubating with 95% ethanol (4°C) for 30 min and subsequently centrifuged at 16,000 *g* for 6 min. The plasma NO concentration depicted in the study is actually the total plasma nitrate and nitrite concentration. The amounts of nitrate and nitrite in the plasma were measured by adding a reducing agent (0.8% VCl_3_ in 1 N HCl) to the purge vessel to convert nitrate to NO, which was stripped from the plasma and drawn into a NO analyzer (Sievers 280 NOA; Sievers Inc., Boulder, CO, USA) by using a helium purge gas. The concentration was determined by comparison with standard solutions of sodium nitrate (Sigma Chemical Co., St Louis, MO, USA).

### Measurement of superoxide production in organs

After thoracic aorta, lung, liver, and kidney were removed from the rat, they were incubated with warmed (37°C), oxygenated (95% O_2_/5% CO_2_) Krebs-HEPES buffer for 10 min. They were transferred to 96-well microplates containing 100 microliters of Krebs-HEPES buffer with 50 microliters of lucigenin (1.25 mM). Then the microplates were placed into a microplate luminometer (Hidex Microplate Luminometer, Turku, Finland) to measure the counts at 10-, 30-, 60-sec intervals for the aorta, liver and kidney, and lung, respectively. All organs were dried in a 95°C oven for 24 h, and the results were expressed as count per second per milligram of tissue dry weight.

### Histopathologic assessment

The specimens of lung and liver were harvested at 6 h and immediately fixed in buffered formaldehyde (10% in phosphate-buffered saline, pH 7.4). The fixed tissues were dehydrated in graded ethanol, embedded in paraffin, and stained with the hematoxylin and eosin reagent for light microscopy. Histological changes were quantitatively as indexes of the severity of polymorphonuclear neutrophil (PMN) infiltration in the lung and liver. Each tissue section was examined under high-power fields by a pathologist in a blinded fashion to give a score from 0 (minimal) to 4 (maximal).

### Immunohistochemistry

For immunohistochemistry, formalin-fixed and paraffin embedded lung sections were prepared. These sections were incubated with primary antibody (5-methylcytosine, 1:1000; GeneTex, Irvine, CA, USA) and then with biotinylated secondary antibody and avidin-biotin-peroxidase complex (Dako, Glostrup, Denmark). The numbers of 5-methylcytosine positive cells were examined in 10 randomly selected fields of the tissues at a magnification of ×400 and expressed values as cells per field. Pax-it quantitative image analysis software (PAXcam, Villa Park, IL, USA) was used as described previously [27].

### Measurement of lung DNMT1 level

At the end of *in viv*o experiment, lungs were collected from animals. Nuclear extract in the lung was obtained by a CNM compartmental protein extraction kit (BioChain Inc., San Francisco, CA, USA) according to the manufacturer’s protocol. Samples containing 10 μg of nuclear extract were processed for analysis. The amount of DNMT1 in nuclear extract was measured with the DNMT1 Assay Kit (Abcam, Cambridge, UK) according to the manufacturer’s instructions.

### Western blot

Nuclear fraction was collected from the lungs of rats by using the CNM compartmental protein extraction kit (BioChain Inc., San Francisco, CA, USA). The experiment was performed by separating 20 μg of protein with 10% sodium dodecyl sulfate-polyacrylamide gel electrophoresis and blotting onto nitrocellulose membrane. The membrane was blocked with primary antibody (DNMT3a, 1:300, purchased from Abcam, Cambridge, UK; DNMT3b, 1:1000, purchased from GeneTex, Irvine, CA, USA; Histone H3, 1:5000, purchased from GeneTex, Irvine, CA, USA), and then reacted with horseradish peroxidase-conjugated goat anti-rabbit IgG (Cell signaling Technology Inc, Danvers, MA, USA). In addition, 100 μg total protein from lung homogenate was used to examine the changes of IL27RA expression. They were separated by 10% sodium dodecyl sulfate-polyacrylamide gel electrophoresis and blotting onto nitrocellulose membrane. The membrane was blocked with primary antibody (IL27RA, 1:2000, purchased from Bio-Rad Laboratories, Philadelphia, USA; β-actin, 1:10000, purchased from BD Transduction Laboratories, Lexington, KY, USA), and then reacted with horseradish peroxidase-conjugated goat anti-rabbit IgG (Cell signaling Technology Inc, Danvers, MA, USA). The protein expressions were recognized by the enhanced chemiluminescence Western blotting reagent (Thermo scientific, Rockford, IL, USA), and then measured by the ImageJ software version 1.46r (National Institutes of Health, Bethesda, MD, USA).

### Microarray analysis

Microarray hybridization and data analysis were performed by Phalanx Biotech Group (Phalanx Biotech Group, Hsinchu, R.O.C., Taiwan). Briefly, total RNA from organ homogenates were extracted using TRIzol reagent, and RNA integrity was determined by Agilent RNA 6000 Nano assay. Raw intensity signals of each microarray were scanned with GenePix Personal 4000B and quantified by using GenePix Pro 4.0 software.

### Bisulfite sequencing

Genomic DNA isolated from rat lung tissue was used to perform bisulfite methylation analysis. We carried out bisulfite conversion of rat genomic DNA by using an EZ DNA methylation kit (Zymo Research, Orange, CA, USA) according to manufacturer’s instruction. Then polymerase chain reaction (PCR) analysis was executed by using primers for rat IL27RA as follow: Forward: 5’-AAGTAGAGGGTTTYGTTGTTTTTTTATAGA-3’; Reverse: 5’- ACCAACTCATAACTCTCAAAAACAAATAAC-3’. PCR amplicon was purified and cloned into RBC TA cloning vector (Real BioTech) and subjected to DNA sequencing.

### Reverse transcription and quantitative PCR

Total RNA was extracted from lung tissues of experimental rats by using the NucleoSpin RNA kit (Macherey-Nagel) and reverse transcription was performed by using Tetro reverse transcriptase (Bioline, London, UK). The cDNAs were then amplified with PowerUp SYBR Green Master Mix (Applied Biosystems) and primers (rat IL27RA, Forward: 5’-TGTTATCAGTGAGCAGTCAAACC-3’, Reverse: 5’-TGAAGTGAAAGGCTGGGACC-3’; rat β-actin, Forward: 5’-TTGGTGGCTCTATCCTGGCCT-3’, Reverse: 5’-AACGCAGCTCAGTAACAGTCCG-3’). qPCR amplification and relative quantifications were performed on the StepOne Real-Time PCR System (Applied Biosystems). Melting curve analysis was conducted to confirm the specificity of PCR.

### Statistical analysis

All data are expressed as mean ± standard error of the mean of *n* determinations, where *n* represents the number of rats studied. Statistical significance between groups was examined through one-way analysis of variance followed by a multiple comparison test (Newman-Keuls test). A *p* value <0.05 was considered statistically significant.

## Results

### Effects of procainamide on hemodynamic parameters

**Fig 1:**
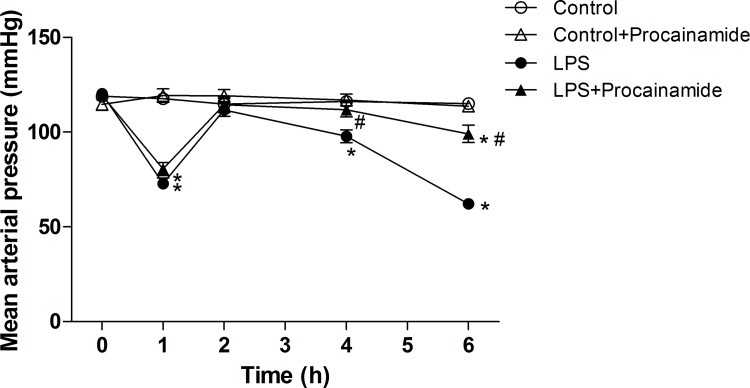
Effects of procainamide on mean arterial pressure in endotoxemic rats. Depicted are the changes during the experimental period in different groups of animals that received saline at time 0 (Control, n = 8), received saline as in Control group and then received procainamide at 1 h (Control + Procainamide, n = 8), received LPS at time 0 (LPS, n = 10), and received LPS at time 0 and then received procainamide at 1 h (LPS + Procainamide, n = 10). Data are expressed as mean ± SEM. *P < 0.05, all versus Control rats; ^#^*P* < 0.05, with versus without procainamide in LPS rats

The animals in the LPS group showed a significant decrease in MAP and pressor responses to NE, and a significant increase in HR during the experimental period. The treatment of LPS rats with procainamide only significantly ameliorated hypotension at 4 and 6 h after LPS (Fig 1), but not tachycardia and vascular hyporesponsiveness to NE induced by LPS (data not shown). In the Control and Control + Procainamide groups, all hemodynamics were not significantly altered during the experimental period.

### Effects of procainamide on blood glucose

**Fig 2:**
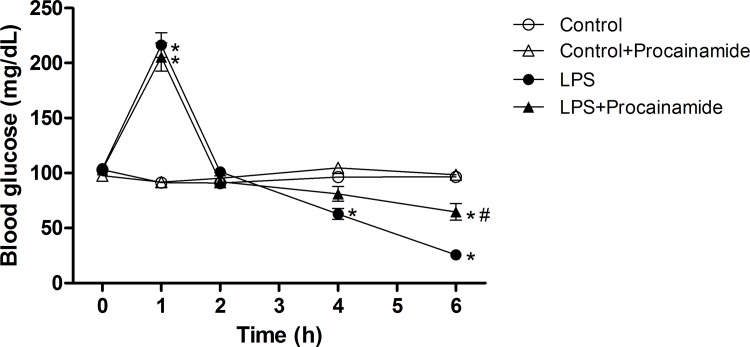
Effects of procainamide on blood glucose in endotoxemic rats. Depicted are the changes during the experimental period in different groups of animals that received saline at time 0 (Control, n = 8), received saline as in Control group and then received procainamide at 1 h (Control + Procainamide, n = 8), received LPS at time 0 (LPS, n = 10), and received LPS at time 0 and then received procainamide at 1 h (LPS + Procainamide, n = 10). Data are expressed as mean ± SEM. *P < 0.05, all versus Control rats; ^#^*P* < 0.05, with versus without procainamide in LPS rats.

The baseline mean values of blood glucose were not significantly different among groups. Hyperglycemia at the early stage (1 h) and hypoglycemia at the late stage (4 and 6 h) were induced by LPS (Fig 2). The treatment of LPS rats with procainamide significantly ameliorated hypoglycemia at 6 h after LPS. In the Control and Control + Procainamide groups, blood glucose level was not significantly changed during the experimental period.

### Effects of procainamide on organ dysfunction/injury caused by LPS

The LPS caused significant increases in plasma levels of ALT, CRE, BUN, and LDH during the experimental period (Table 1). The increases of ALT, CRE, and LDH, but not BUN, caused by LPS at 6 h were significantly attenuated by procainamide administration (Table 1). However, all organ function indexes were not significantly changed during the experimental period in the Control and Control + Procainamide groups.

**Table 1:** Effects of procainamide on organ function/injury in LPS-induced endotoxemic rats.

	0 h	1 h	2 h	4 h	6 h
**ALT (U/L)**					
Control	22.63 ± 2.14	18.13 ± 1.90	17.25 ± 1.73	16.88 ± 1.58	14.75 ± 1.45
Control + Procainamide	21.25 ± 1.24	18.50 ± 1.72	14.75 ± 1.56	16.63 ± 1.83	18.63 ± 2.24
LPS	19.70 ± 1.39	56.80 ± 17.38	61.50 ± 17.81	99.70 ± 28.74*	208.80 ± 46.20*
LPS + Procainamide	20.70 ± 2.75	45.50 ± 13.83	46.30 ± 14.52	73.30 ± 20.55	110.10 ± 34.46*†
**CRE (mg/dL)**					
Control	0.20 ± 0	0.20 ± 0	0.20 ± 0	0.20 ± 0	0.20 ± 0
Control + Procainamide	0.20 ± 0	0.20 ± 0	0.20 ± 0	0.20 ± 0	0.20 ± 0
LPS	0.20 ± 0	0.26 ± 0.02	0.27 ± 0.03	0.52 ± 0.05*	0.97 ± 0.10*
LPS + Procainamide	0.20 ± 0	0.26 ± 0.02	0.26 ± 0.03	0.43 ± 0.05*	0.53 ± 0.08*†
**BUN (mg/dL)**					
Control	19.21 ± 0.81	17.93 ± 0.99	17.65 ± 0.95	17.13 ± 0.96	16.68 ± 1.06
Control + Procainamide	19.94 ± 0.55	19.65 ± 1.45	18.26 ± 1.82	19.18 ± 1.78	18.99 ± 1.73
LPS	19.52 ± 0.47	31.91 ± 0.53*	38.04 ± 0.99*	63.16 ± 1.87*	81.34 ± 1.55*
LPS + Procainamide	20.30 ± 0.97	32.05 ± 1.29*	36.39 ± 2.36*	56.83 ± 3.69*	74.64 ± 6.22*
**LDH (U/L)**					
Control	94.00 ± 9.05	92.50 ± 12.57	71.50 ± 9.34	79.00 ± 11.53	65.38 ± 6.95
Control + Procainamide	86.13 ± 8.37	86.00 ± 6.83	85.13 ± 9.06	131.13 ± 56.50	130.50 ± 52.04
LPS	81.80 ± 8.43	358.40 ± 44.86	471.40 ± 58.34	1110.00 ± 189.04*	2523.00 ± 372.91*
LPS + Procainamide	80.50 ± 4.16	271.50 ± 55.25	505.30 ± 84.61	1006.30 ± 380.24*	1448.90 ± 657.82*†

Depiction are the changes in plasma levels of alanine aminotransferase (ALT), creatinine (CRE), blood urea nitrogen (BUN), and lactate dehydrogenase (LDH) during the experimental period in different groups of animals. Control, rats were given saline at time 0 (n = 8); Control + Procainamide, rats were given saline as in Control group and then given procainamide at 1 h (n = 8); LPS, rats were given LPS at time 0 (n = 10); LPS + Procainamide, rats were given LPS at time 0 and then given procainamide at 1 h (n = 10). Data are expressed as mean ± SEM.

*P < 0.05, all versus Control rats

†*P* < 0.05, with versus without procainamide in LPS rats

### Effects of procainamide on plasma NO levels

**Fig 3:**
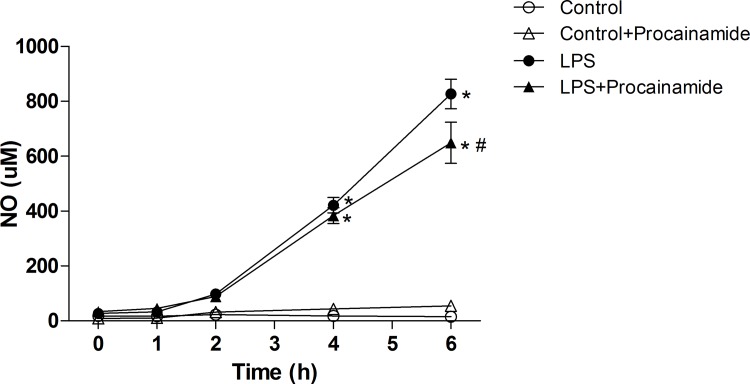
Effects of procainamide on plasma nitric oxide (NO) in endotoxemic rats. Depicted are the changes during the experimental period in different groups of animals that received saline at time 0 (Control, n = 5), received saline as in Control group and then received procainamide at 1 h (Control + Procainamide, n = 5), received LPS at time 0 (LPS, n = 5), and received LPS at time 0 and then received procainamide at 1 h (LPS + Procainamide, n = 5). Data are expressed as mean ± SEM. *P < 0.05, all versus Control rats; ^#^*P* < 0.05, with versus without procainamide in LPS rats.

In the Control and Control + Procainamide groups, no significant increases in plasma NO levels were detected during the experimental period (Fig 3). In contrast, animals that received LPS had significantly increased plasma NO levels at 4 and 6 h, and this increment of plasma NO at 6 h after LPS was attenuated by procainamide (Fig 3).

### Effects of procainamide on superoxide levels in organs

**Fig 4:**
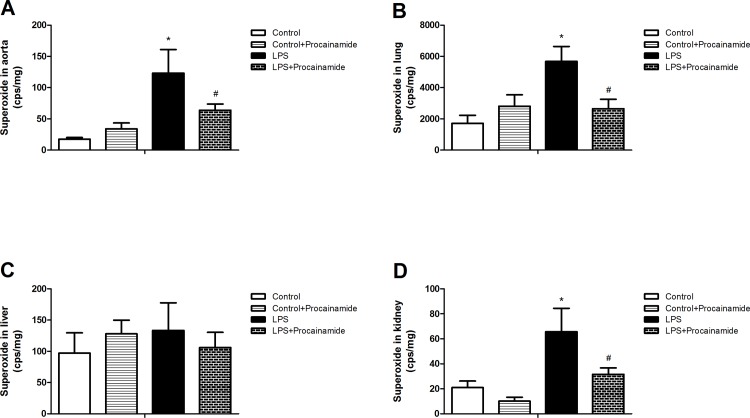
**Effects of procainamide on superoxide levels in (A) aortas, (B) lungs, (C) livers, and (D) kidneys obtained from endotoxemic rats.** Depicted are changes at the end of experiments (at 6 h) in different groups of animals that received saline at time 0 (Control, n = 6), received saline as in Control group and then received procainamide at 1 h (Control + Procainamide, n = 6), received LPS at time 0 (LPS, n = 6), and received LPS at time 0 and then received procainamide at 1 h (LPS + Procainamide, n = 6). Data are expressed as mean ± SEM. *P < 0.05, all versus Control rats; ^#^*P* < 0.05, with versus without procainamide in LPS rats.

The basal production of superoxide was detectable in aortas (Fig 4A), lungs (Fig 4B), livers (Fig 4C), and kidneys (Fig 4D) obtained from the Control group. In the Control + Procainamide groups, there were no significant changes of superoxide levels in these tissues. However, the animals that received LPS showed significant increases in the production of superoxide in the aorta (Fig 4A), lung (Fig 4B), and kidney (Fig 4D), whereas LPS had no significant effect on superoxide levels in the liver (Fig 4C). In LPS + Procainamide rats, the elevation of superoxide levels in the aorta, lung, and kidney were significantly inhibited by procainamide (Fig 4A, 4B and 4D).

### Effects of procainamide on neutrophil infiltration in organs

**Fig 5:**
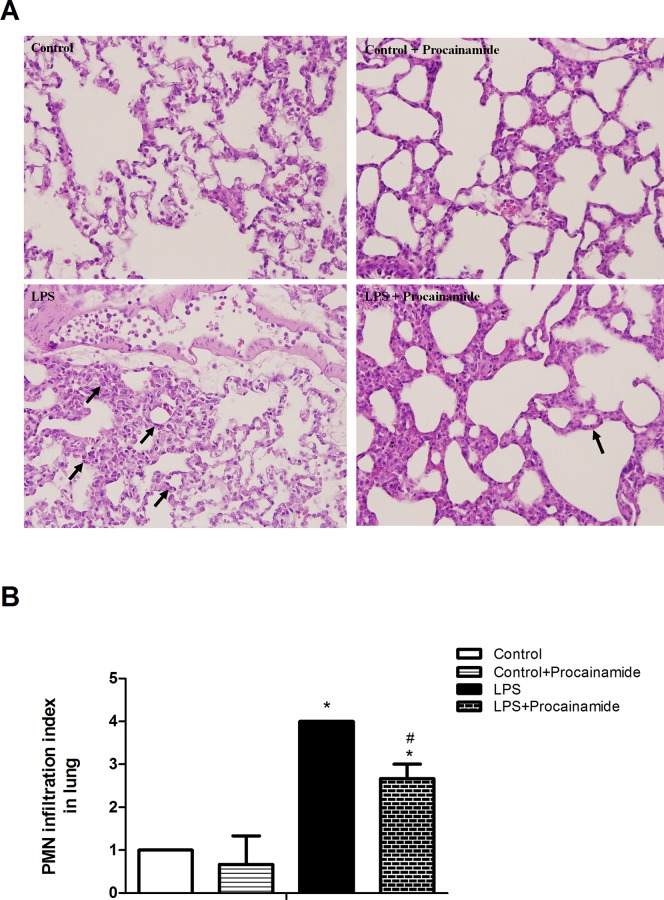
**(A) Representative histopathologic features and (B) polymorphonuclear neutrophil (PMN) infiltration index of lung tissue sections obtained from endotoxemic rats.** Depicted are changes in PMN infiltration index at the end of experiment (at 6 h) in different groups of animals that received saline at time 0 (Control, n = 3), received saline as in Control group and then received procainamide at 1 h (Control + Procainamide, n = 3), received LPS at time 0 (LPS, n = 3), and received LPS at time 0 and then received procainamide at 1 h (LPS + Procainamide, n = 3). Data are expressed as mean ± SEM. *P < 0.05, all versus Control rats; ^#^*P* < 0.05, with versus without procainamide in LPS rats. Arrows represent neutrophil infiltration. Original magnification x 200.

**Fig 6:**
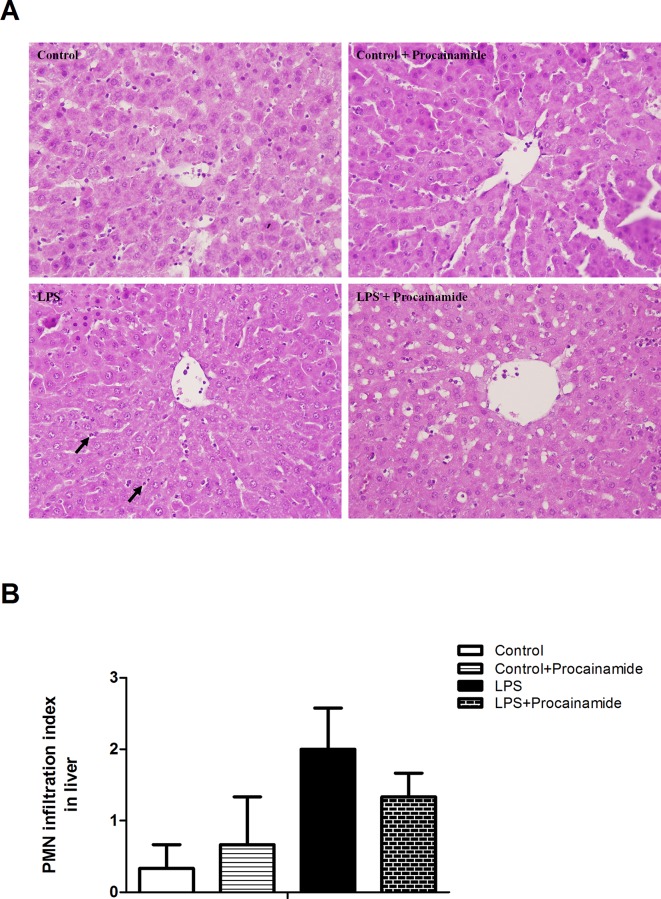
**(A) Representative histopathologic features and (B) polymorphonuclear neutrophil (PMN) infiltration index of liver tissue sections obtained from endotoxemic rats.** Depicted are changes in PMN infiltration index at the end of experiment (at 6 h) in different groups of animals that received saline at time 0 (Control, n = 3), received saline as in Control group and then received procainamide at 1 h (Control + Procainamide, n = 3), received LPS at time 0 (LPS, n = 3), and received LPS at time 0 and then received procainamide at 1 h (LPS + Procainamide, n = 3). Data are expressed as mean ± SEM. *P < 0.05, all versus Control rats; ^#^*P* < 0.05, with versus without procainamide in LPS rats. Arrows represent neutrophil infiltration. Original magnification x 400.

In the Control and Control + Procainamide groups, light microscopy only showed a small amount of neutrophil infiltration in the lung (Fig 5) and liver (Fig 6). Not surprisingly, the animals that received LPS showed an overt neutrophil infiltration in the lung (Fig 5) but not in the liver (Fig 6). However, the treatment of LPS rats with procainamide significantly improved neutrophil infiltration in the lung (Fig 5).

### Effects of procainamide on 5-methylcytosine levels in the lung

**Fig 7:**
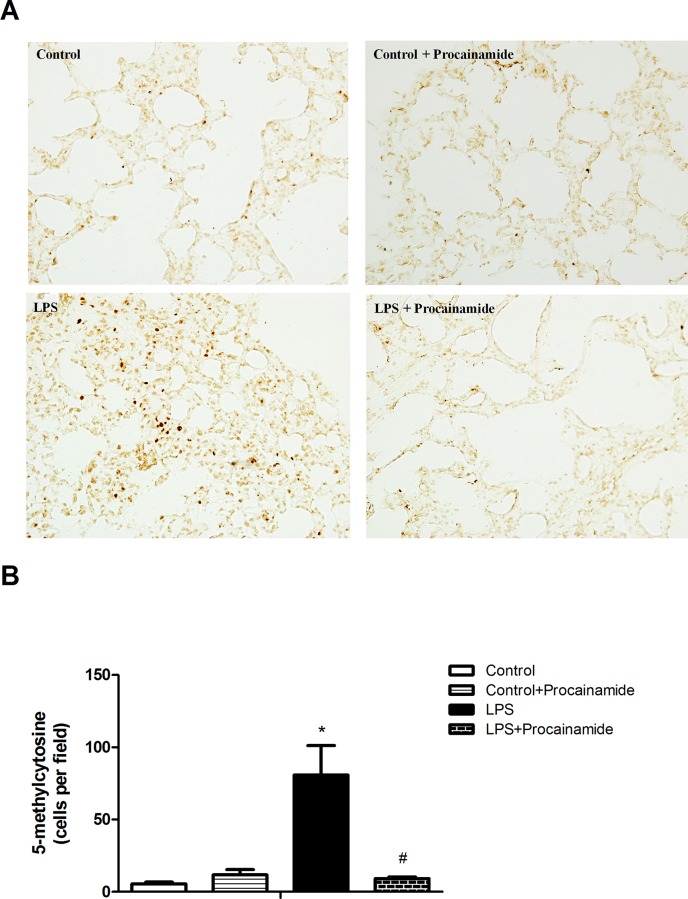
**(A) Detection of 5-methylcytosine by immunohistochemistry and (B) the numbers of 5-methylcytosine positive cells in the lungs obtained from different groups of animals.** Depicted are changes in the numbers of lung 5-methylcytosine positive cells at the end of experiment (at 6 h) in different groups of rats that received saline at time 0 (Control, n = 3), received saline as in Control group and then received procainamide at 1 h (Control + Procainamide, n = 3), received LPS at time 0 (LPS, n = 3), and received LPS at time 0 and then received procainamide at 1 h (LPS + Procainamide, n = 3). Data are expressed as mean ± SEM. *P < 0.05, all versus Control rats; ^#^*P* < 0.05, with versus without procainamide in LPS rats. Original magnification x 400.

The basal level of 5-methylcytosine was observed in the lung of Control group (Fig 7). In the Control + Procainamide group, there were no significant changes in 5-methylcytosine levels when compared with the Control group (Fig 7). The numbers of 5-methylcytosine positive cells were significantly increased in the lungs of rats with endotoxic shock (Fig 7). However, treatment of endotoxemic rats with procainamide significantly diminished the elevation of 5-methylcytosine levels (Fig 7).

### Effects of procainamide on DNMT1 levels in the lung

**Fig 8:**
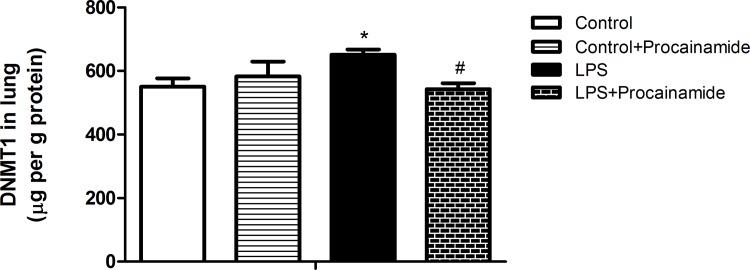
Effects of procainamide on DNMT1 levels in the lungs obtained from different groups of animals. Depicted are changes in lung DNMT1 levels at the end of experiment (at 6 h) in different groups of rats that received saline at time 0 (Control, n = 4), received saline as in Control group and then received procainamide at 1 h (Control + Procainamide, n = 4), received LPS at time 0 (LPS, n = 7), and received LPS at time 0 and then received procainamide at 1 h (LPS + Procainamide, n = 7). Data are expressed as mean ± SEM. *P < 0.05, all versus Control rats; ^#^*P* < 0.05, with versus without procainamide in LPS rats.

The basal level of DNMT1 was detectable in the lung from the Control group, and it was similar to that in the Control + Procainamide group (Fig 8). However, significant elevations of DNMT1 levels in the lung were observed at 6 h after rats treated with LPS, and this elevation was attenuated by procainamide in LPS + Procainamide rats (Fig 8).

### Effects of procainamide on the expressions of DNMT3a and DNMT3b

**Fig 9:**
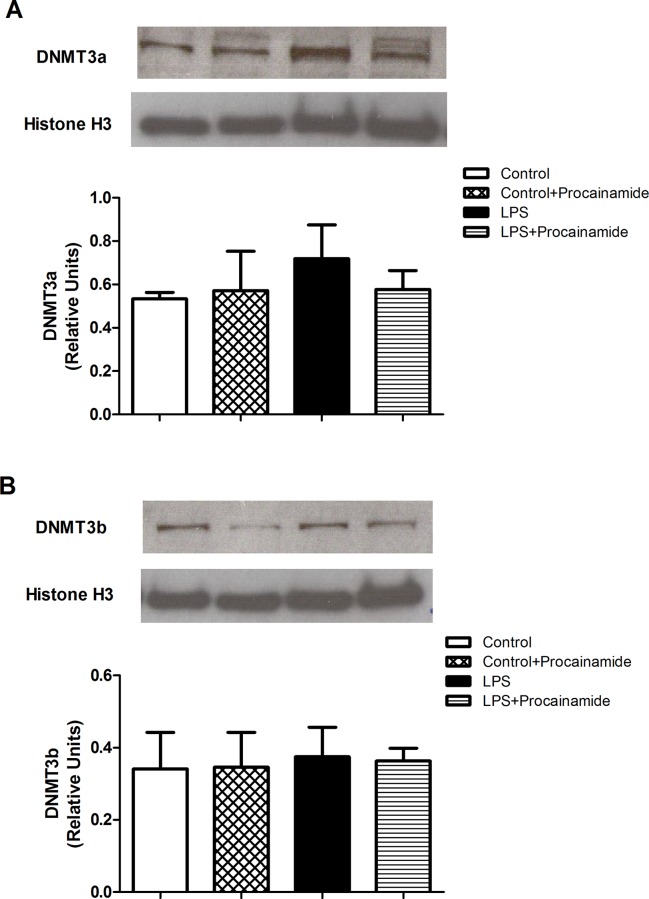
**Effects of procainamide on the expressions of (A) DNMT3a and (B) DNMT3b in the lungs obtained from different groups of animals.** Depicted are changes in lung DNMT3a and DNMT3b expressions at the end of experiment (at 6 h) in different groups of rats that received saline at time 0 (Control, n = 3), received saline as in Control group and then received procainamide at 1 h (Control + Procainamide, n = 3), received LPS at time 0 (LPS, n = 3), and received LPS at time 0 and then received procainamide at 1 h (LPS + Procainamide, n = 3). Data are expressed as mean ± SEM. *P < 0.05, all versus Control rats; ^#^*P* < 0.05, with versus without procainamide in LPS rats

In the LPS group, the expressions of DNMT3a and DNMT3b were similar to the Control group (Fig 9A and 9B). In addition, there was no significant difference in the expressions of DNMT3a and DNMT3b between the LPS and LPS + Procainamide groups (Fig 9A and 9B).

### Effects of hydralazine on hypotension, hypoglycemia, and organ dysfunction

**Fig 10:**
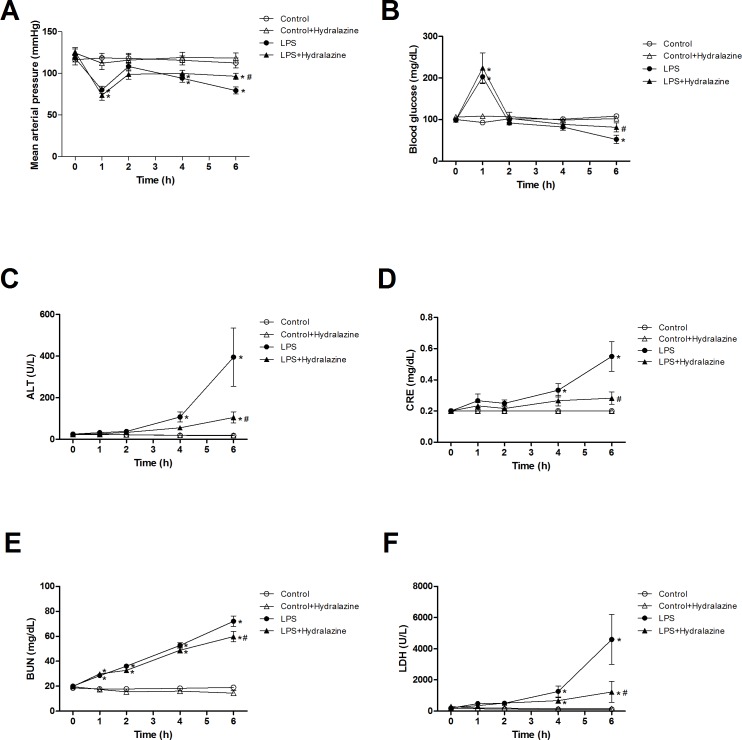
**Effects of hydralazine on (A) mean arterial pressure, (B) blood glucose, (C) alanine aminotransferase (ALT), (D) creatinine (CRE), (E) blood urea nitrogen (BUN), and (F) lactate dehydrogenase (LDH) in endotoxemic rats.** Depicted are the changes during the experimental period in different groups of animals that received saline at time 0 (Control, n = 6), received saline as in Control group and then received hydralazine at 0.5 h (Control + Hydralazine, n = 6), received LPS at time 0 (LPS, n = 6), and received LPS at time 0 and then received hydralazine at 0.5 h (LPS + Hydralazine, n = 6). Data are expressed as mean ± SEM. *P < 0.05, all versus Control rats; ^#^*P* < 0.05, with versus without hydralazine in LPS rats.

The administration of LPS rats with hydralazine significantly improved hypotension and hypoglycemia at 6 h after LPS (Fig 10A and 10B), but hydralazine had no effects on tachycardia and vascular hyporesponsiveness to NE induced by LPS (data not shown). In the Control and Control + Hydralazine groups, hemodynamics and blood glucose levels were not significantly altered during the experimental period. In addition, the elevations of ALT, CRE, BUN, and LDH caused by LPS at 6 h were significantly attenuated by hydralazine administration (Fig 10C–10F). However, no significant increases in organ functional indexes were detected during the experimental period in the Control and Control + Hydralazine groups.

### Effects of procainamide on survival rate

No mortality was observed within 6 h in both Control and Control + Procainamide groups (Table 2). The 3-h, 4-h, 5-h, and 6-h survival rates of animals that received LPS were 78%, 59%, 31%, and 31%, respectively (Table 2). Treatment of LPS rats with procainamide increased the survival rates at 3–6 h (Table 2).

**Table 2:** Effects of procainamide on survival rate in LPS-induced endotoxemic rats.

Groups	1-h Survival Rate (%)	2-h Survival Rate (%)	3-h Survival Rate (%)	4-h Survival Rate (%)	5-h Survival Rate (%)	6-h Survival Rate (%)
Control	100 (8/8)	100 (8/8)	100 (8/8)	100 (8/8)	100 (8/8)	100 (8/8)
Control + Procainamide	100 (8/8)	100 (8/8)	100 (8/8)	100 (8/8)	100 (8/8)	100 (8/8)
LPS	100 (32/32)	100 (32/32)	78 (25/32)	59 (19/32)	31 (10/32)	31 (10/32)
LPS + Procainamide	100 (24/24)	100 (24/24)	92 (22/24)	71 (17/24)	42 (10/24)	42 (10/24)

Depiction of the changes in survival rate during the experimental period in different groups of animals. Control, rats were given saline at time 0 (n = 8); Control + Procainamide, rats were given saline as in Control group and then given procainamide at 1 h (n = 8); LPS, rats were given LPS at time 0 (n = 32); LPS + Procainamide, rats were given LPS at time 0 and then given procainamide at 1 h (n = 24). Data are expressed as percentage of rats survived at the observed time point.

### Effects of procainamide on gene expression in the lung

**Fig 11:**
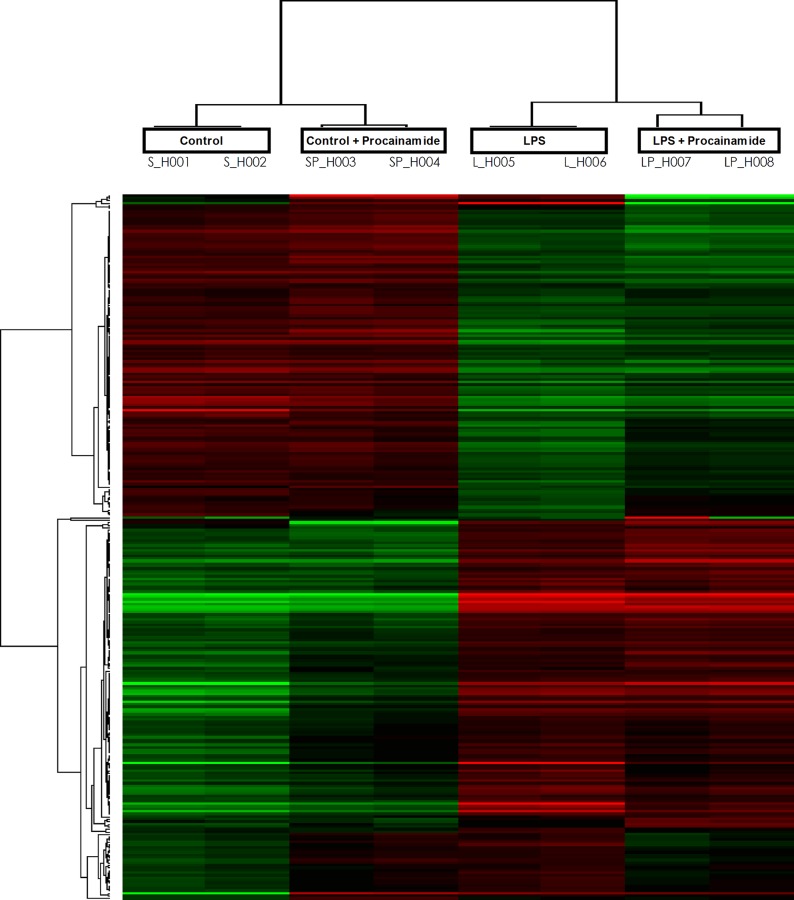
Clustered display of microarray data from lung tissue. Depicted are the changes in different groups of animals that received saline at time 0 (Control), received saline as in Control group and then received hydralazine at 0.5 h (Control + Hydralazine), received LPS at time 0 (LPS), and received LPS at time 0 and then received hydralazine at 0.5 h (LPS + Hydralazine). Up-regulated and down-regulated genes are symbolized in red and green colors, respectively.

We used gene microarray to detect changes in the expression level of all genes. For advanced data analysis, intensity data were pooled and calculated to identify differentially expressed genes based on the threshold of fold change and p-value. The correlation of expression profiles between samples and treatment conditions was demonstrated by unsupervised hierarchical clustering analysis. Clustering was performed to visualize the correlations among the replicates and vary sample conditions. A subset of differential genes was selected for clustering analysis. An intensity filter was used to select genes where the difference between the maximum and minimum intensity values exceeds 50,000 among all microarrays. For this microarray project, the number of genes clustered was 276 (Fig 11).

The analysis identified 3,240 transcripts differentially expressed in lung samples between LPS and Control group. Of these transcripts, 1,625 genes were up-regulated and 1,615 genes were down-regulated in the LPS group with a fold change of at least 1.5 and a p-value of less than 0.05 (Table 3). In addition, there were 1,074 transcripts differentially expressed in lung samples between LPS + Procainamide and LPS group. Of these transcripts, 566 genes were up-regulated and 508 genes were down-regulated in the LPS + Procainamide group (Table 3). One hundred forty-one genes were overlapping between down-regulated genes in the LPS group compared with Control group and up-regulated genes in the LPS + Procainamide group compared with LPS group. Of the 141 overlap genes, IL27RA was included in the subsequent methylation experiment as it was known to be relevant to the anti-inflammatory signaling.

**Table 3:** Effects of procainamide on gene expression in LPS-induced endotoxemic rats.

Group comparison	Up-regulated gene number	Down-regulated gene number
Control + Procainamide v.s. Control	412	200
LPS v.s. Control	1625	1615
LPS + Procainamide v.s. LPS	566	508

Depiction of the changes in gene expression in different groups of animals. Control, rats were given saline at time 0; Control + Procainamide, rats were given saline as in Control group and then given procainamide at 1 h; LPS, rats were given LPS at time 0; LPS + Procainamide, rats were given LPS at time 0 and then given procainamide at 1 h.

### Effects of procainamide on DNA methylation pattern of IL27RA promoter

**Fig 12:**
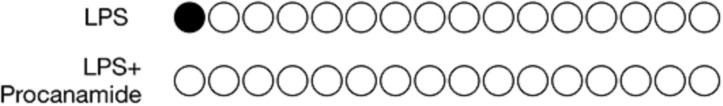
Effects of procainamide on IL27RA methylation in the lung of endotoxemic rat. The 532 bp amplicon corresponds to the rat IL27RA promoter region from -401 to +131 (from the transcription start site of IL27RA). Sixteen CpG dinucleotides within the CpG islands were examined and denoted as open circle (unmethylated) and closed circle (methylated).

Sixteen CpG dinucleotides within the CpG islands of IL27RA promoter were analyzed in the LPS and LPS + Procainamide groups. IL27RA promoter of the lung tissue obtained from LPS rat showed methylation in the first CpG dinucleotides (Fig 12). However, there was no methylated CpG dinucleotides in IL27RA promoter of the lung tissue obtained from LPS rat treated with procainamide (Fig 12).

### Effects of procainamide on IL27RA mRNA and protein levels in the lung

**Fig 13:**
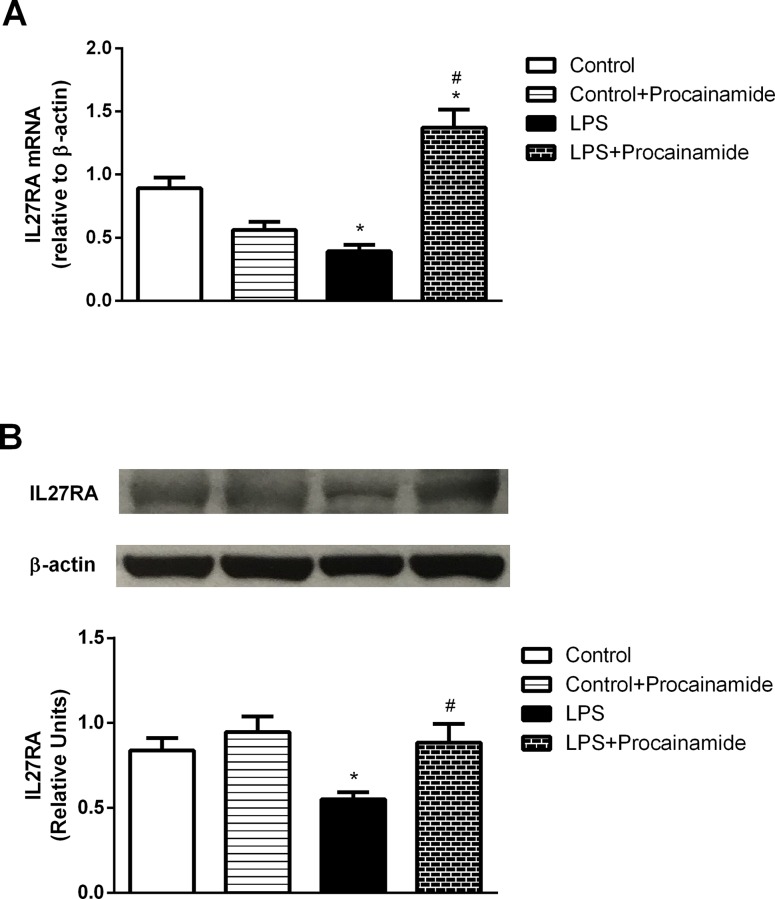
**Effects of procainamide on the expressions of (A) IL27RA mRNA and (B) IL27RA protein in the lungs obtained from different groups of animals.** Depicted are changes in lung IL27RA mRNA and protein expressions at the end of experiment (at 6 h) in different groups of rats that received saline at time 0 (Control, n = 4), received saline as in Control group and then received procainamide at 1 h (Control + Procainamide, n = 4), received LPS at time 0 (LPS, n = 4), and received LPS at time 0 and then received procainamide at 1 h (LPS + Procainamide, n = 4). Data are expressed as mean ± SEM. *P < 0.05, all versus Control rats; ^#^*P* < 0.05, with versus without procainamide in LPS rats

The levels of IL27RA mRNA were decreased in the lungs of rats with endotoxic shock (Fig 13A). Administration of endotoxemic rats with procainamide significantly increased IL27RA mRNA levels (Fig 13A). However, there was no significant difference in IL27RA mRNA levels between Control and Control + Procainamide groups (Fig 13A).

Significant reduction of IL27RA protein expression was also observed in the lungs obtained from the rats treated with LPS (Fig 13B). In LPS + Procainamide rats, the expressions of IL27RA protein were significantly up-regulated (Fig 13B). However, the expressions of IL27RA protein in the Control group were similar to those in the Control + Procainamide group (Fig 13B).

## Discussion

In this study, LPS-treated rats revealed circulatory failure, multiple organ dysfunction, and high mortality rate, as seen in patients with endotoxic shock. The increased levels of DNMT1 and 5-methylcytosine occurred and resulted in the elevation of PMN infiltration and superoxide production in rats with endotoxic shock. Treatment with procainamide improved severe hypotension, hypoglycemia, multiple organ dysfunction, and high mortality rate in endotoxemic animals. In addition, procainamide reduced IL27RA methylation and augmented IL27RA expression in the lung of endotoxemic rat. Moreover, another inhibitor of DNMT (i.e. hydralazine) had similar effects as procainamide had, e.g. alleviation of hypotension, hypoglycemia, and multiple organ dysfunction. Thus, we suggest that the beneficial effects of procainamide could be attributed to the attenuation of overt (i) DNA methylation, (ii) PMN accumulation, (iii) superoxide production, and (iv) NO formation in LPS-induced endotoxemic rats.

DNA methylation regulated by DNMTs occurs primarily at the 5-position of cytosine in the CpG dinucleotide [28]. An increasing number of studies support that DNA methylation modifications linked to the transcription of host protective genes are relatively common during pathogen infections [6, 29]. Zhang et al. have shown that aberrant DNA methylation occurs in rat injured lungs induced by LPS [11]. In addition, the changes of DNA methylation in the calcitonin-related polypeptide alpha gene are observed in preterm infants with bacterial sepsis [30]. Indeed, our results also showed that LPS enhanced the expression of DNMT1 and increased the level of 5-methylcytosine in the lung, indicating a crucial role of DNA methylation in the progression of endotoxic shock. Moreover, the administration of LPS rats with procainamide attenuated DNA hypermethylation in the lungs of rats with endotoxic shock. In order to identify changes in the expression level of putative candidate genes during endotoxemia in rats and after treatment with procainamide, gene microarray was used to detect changes in the expression level. We found that IL27RA was down-regulated in the LPS group and up-regulated in the LPS + Procainamide group. IL27RA has been demonstrated to exert anti-inflammatory function in several models of inflammation [31, 32]. IL27RA-deficient mice are prone to enhanced expression of Th17-related cytokine and exacerbation of mucus secretion in the lung following respiratory syncytial virus infection [33]. Moreover, impaired IL27RA signaling leads to massive pulmonary infiltration, severe lung pathology, and higher mortality after influenza virus challenge [34]. In our study, the levels of IL27RA mRNA and protein were diminished in the lungs of endotoxemic rats, indicating that IL27RA signaling is damaged after LPS challenge. This could result in less protection from exaggerated inflammation. However, treatment with procainamide reduced IL27RA methylation and augmented IL27RA expressions in the lungs of endotoxemic rats. Therefore, these results suggest that procainamide is a promising therapy to reverse abnormal DNA methylation status and up-regulate protective gene expression in endotoxemia.

Microbial products induce dysregulated activation of innate immune system to cause detrimental consequences in patients with sepsis [1, 35]. Some previous studies show the initiation and progression of inflammation in inflammatory disorders are regulated by DNA methylation [36–38]. We used histological assessment in this study because we not only wanted to evaluate the effects of procainamide on neutrophil filtration but also wanted to examine the effects of procainamide on tissue injury such as interstitial edema in the lung or necrosis in the liver. Our study demonstrated that DNMT1-mediated DNA hypermethylation enhanced neutrophil infiltration in the lungs of rats with endotoxic shock. Overt PMN accumulation in the lung was significantly improved after treating endotoxemic animals with DNMT1 inhibitor procainamide. Similar anti-inflammatory effects are observed when other DNMT inhibitors such as 5-aza-dC and DNMT1 siRNA are used to diminish oscillatory shear stress-induced endothelial inflammation [37]. In addition, macrophage inflammation, migration, and adhesion in atherosclerotic plaques are inhibited by 5-aza-dC [15]. These findings further support that procainamide diminished endotoxin-induced leukocyte accumulation via alleviating DNA hypermethylation. We suggest both circulating leukocytes and structural cells of the lung could be affected in our study according to some previous studies that demonstrate the protective genes are abnormally methylated in leukocytes and lung cells during stress conditions [11, 15].

The imbalance in redox state elicits oxidative stress that results in mitochondrial dysfunction and tissue damage in sepsis [39, 40]. Excessive DNA methylation of antioxidant genes leads to the increase of oxidative stress in many diseases [17, 41]. We found that both DNA methylation and superoxide production increased in the organs of animals with endotoxic shock in this study. Administration of LPS rats with procainamide alleviated DNA hypermethylation and superoxide overproduction, indicating the reduction of oxidative stress in septic animals treated with procainamide is due to its demethylating effects. Moreover, the effects of DNMT inhibitors on the rescue of antioxidant gene expression and the decrease of ROS production are observed in osteoarthritis chondrocytes and lung adenocarcinoma cells [16, 18]. Taken together, procainamide may serve as a therapeutic drug for the diseases caused by oxidative stress.

Excessive NO production in sepsis is detrimental because NO and its byproduct peroxynitrite elicit severe hypotension and multiple organ dysfunction [42–44]. Neutralization of the increased NO can prevent the progression of septic shock. Indeed, procainamide significantly lowered the plasma NO levels, leading to the attenuation of hypotension, multiple organ dysfunction, and mortality rate in rats with endotoxic shock. The protective actions of procainamide against nephrotoxicity and hepatotoxicity have been reported in animals treated with cisplatin [45, 46]. On the other hand, previous studies suggest that endotoxin, cytokines or ROS can stimulate inducible NO synthase expression to enhance NO production in various cells [47, 48]. The changes in DNA methylation status have been associated with the development of inflammation and oxidative stress. In addition, we used another DNMT inhibitor, hydralazine, to provide similar evidence in revealing the correlation between DNA methylation and sepsis. The results showed that hydralazine also improved hypotension, hypoglycemia, and multiple organ dysfunction of LPS-treated rats. Therefore, the beneficial effects of procainamide on endotoxic shock are potentially related to the inhibition of DNA methylation in the lung, secondary to the attenuation of PMN infiltration, superoxide production, and NO formation. This is the first demonstration indicating that procainamide treatment protects against circulatory failure and organ injury caused by endotoxin.

To summarize our results, procainamide prevented severe hypotension, hypoglycemia, and multiple organ dysfunction, leading to an increase in the survival rate of LPS-treated rats. Possible mechanisms contributing to the beneficial effects of procainamide include the suppression of DNA methylation, reduction of organ superoxide production, attenuation of lung neutrophil infiltration, and inhibition of plasma NO formation. However, other therapeutic mechanisms of procainamide need to be explored. Our study indicates this old drug could have new potential therapeutic uses in infectious diseases, in particular, associated with endotoxemia.
